# Idiopathic Edema: A Case Report

**DOI:** 10.7759/cureus.5250

**Published:** 2019-07-27

**Authors:** Nikhila Veluri, Karun Badwal

**Affiliations:** 1 General Medicine, American University of Integrative Sciences, St. Michael, BRB; 2 Internal Medicine: Geriatrics, Mayo Clinic, Rochester, USA

**Keywords:** edema, idiopathic edema, fluid retention, periodic edema, cyclical edema

## Abstract

Edema occurs when excess fluid is retained by interstitial tissue and is associated with several systemic conditions. The diagnosis of edema is generally made by physical and clinical assessment, and diuretics are the common treatment. Idiopathic edema predominantly affects women and occurs in patients who do not have any other pre-existing systemic problems. The etiology behind idiopathic edema has yet to be determined. We present a case of a 46-year-old woman diagnosed with idiopathic edema.

## Introduction

Edema is the excess of fluid outflow into the interstitium and is most commonly caused by an increase in hydrostatic capillary pressure, a decrease in oncotic capillary pressure, an increase in capillary permeability or an increase in interstitial oncotic pressure [1]. Idiopathic edema is a state which primarily affects women in the absence of hepatic, renal, and cardiac diseases [2,3]. Diagnosis is generally made by physical and clinical assessment. A variety of conditions have been associated with edema. The workup for these conditions includes measuring the brain natriuretic peptide, creatinine, albumin, liver enzymes and d-dimer assay. Imaging techniques routinely used are duplex or venous ultrasonography. Yet, despite these various tests, edema remains to be a diagnosis of exclusion. Edema may persist over time or develop periodically (idiopathic cyclic edema). There is usually generalized swelling of the hands, legs, and face. A patient with edema may generally experience changes in weight throughout the day. This report describes a case of a 46-year-old woman diagnosed with idiopathic edema.

## Case presentation

A 46-year-old white woman presented to the Hypertension and Nephrology Clinic with significant weight gain and edema. She reported concerns regarding her weight gain of 70 pounds over the last six months. She stated that she is an active person and believed her weight gain was primarily due to fluid retention. She received laparoscopic adjustable gastric banding in 2003, followed by an abdominoplasty in 2016. She reported she was doing well and lost a significant amount of weight after her surgeries until six months prior to presentation. She consulted her surgeon and endocrinologist, both of whom reassured her that her gastric band was intact without any complications. Since August of 2018, she has been taking furosemide 20 mg by mouth, once daily along with spironolactone 25 mg by mouth once daily. The patient has not found any significant improvement with these medications and reported oliguria. She denied any prior history of diabetes, hypertension, or kidney stones.

Her past medical history was significant for chronic idiopathic abdominal pain, varicose veins, and edema. Her surgical history begins in 1978 for a tonsillectomy, laparoscopic adjustable gastric banding and cholecystectomy in 2003, a bariatric bypass in 2009, a colonoscopy in 2013 followed by an abdominoplasty in 2016, and an esophagogastroduodenoscopy in 2018. Her family history is only significant for unspecified cancer in her father. The patient reports an allergy to latex and denies alcohol or tobacco use. She admits to smoking cannabis on rare social occasions. Her laboratory results and vital signs were unremarkable. However, her BMI was 33.39 kg/m^2^ as she was 68 inches tall and weighed 219 pounds.

She was referred to the Hypertension and Nephrology Clinic to assess whether her edema was due to any renal abnormalities or conditions. Patient’s lab work demonstrated that her kidney function was normal as all her electrolytes and creatinine levels were within the reference range. Given our patient had no heart failure, liver dysfunction, kidney dysfunction, malignancies, or protein malnutrition, she was diagnosed with idiopathic edema. This patient was referred to a lymphedema clinic for further evaluation.

## Discussion

The four most common causes of edema include an increase in hydrostatic capillary pressure (commonly seen in heart failure patients), a decrease in oncotic capillary pressure (common to patients with liver disorders, nephrotic syndromes or malnutrition), an increase in capillary permeability (as seen with damage from burns or inflammation) or an increase in interstitial oncotic pressure (associated with leukocyte-mediated injury and endothelial cell growth) [2-4]. When the interstitial fluid surpasses the ability of the lymphatic system to return it to circulation, edema will be formed due to the increase in interstitial oncotic pressure. Thus, impaired lymphatic drainage is another cause of edema, often called lymphedema. Parasitic infections of lymph nodes, removal or irradiation of lymph nodes due to malignancy, and lack of muscular activity can lead to lymphedema [4].

Idiopathic edema is a condition predominantly found in women and occurs in patients who do not have any other pre-existing systemic problems. Idiopathic edema is generally diagnosed clinically, but several confirmation tests are available to aid in the diagnosis. One test for idiopathic edema is conducted by having the patient check her weight in the mornings and evenings on an empty bladder, before having any food or fluids. A weight gain of greater than 0.7 kg is indicative of idiopathic edema [5]. Another such test, the water load test, includes having the patient drink 20 mL/kg body weight (maximum of 1500 mL) of non-iced water over 20 minutes in the mornings between 7:30 AM to 9:00 AM. Urine is collected on an hourly basis, starting an hour before the first oral fluid load and ending four hours afterward. During these four hours, patients must be either standing or walking slowly on the first day. On the second day, the patient repeats the same fluid oral load and urine collection but must be in the recumbent position during the four hours. Everyone with idiopathic edema exhibits urine excretion of less than 55% of the water load in the upright position while excreting more than 65% of the water load while in the recumbent position [5].

Shahmirzai and Firouzifar found that patients with sleep apnea (i.e., recurrent episodic stoppage of breathing during sleep) also had an association with idiopathic leg edema. They found a slightly higher trend in female patients. Men were more prone at younger ages [6]. Obstructive sleep apnea (OSA) can be a partial or complete narrowing of the pharyngeal airway [7]. Obesity has been a risk factor for OSA as patients have enlargement of soft tissue structures within and around the airway causing substantial pharyngeal airway narrowing [8]. Blankfield et al. reported a significant association between idiopathic edema and OSA, particularly in women (P = 0.02) [9]. Combining findings from both studies, it is possible that our patient’s generalized edema could be secondary to OSA. This can be further investigated with a sleep study. Although our patient reported no symptoms of OSA, increased daytime sleepiness, fatigue, irritability or morning headaches, the possibility of sleep apnea-induced edema could not be ruled out given her BMI of 33.39 kg/m^2^ and history of obesity.

Diuretics have also been linked to idiopathic edema. Extended use of diuretics causes progressive resistance to and dependence on diuretics [10,11]. While our patient reported using diuretics for seven months, it is possible she had been using diuretics for longer than reported. Abrupt withdrawal diuretic use can cause life-threatening conditions; therefore, a slow weaning method must be conducted. In addition to decreased diuretic dosage titration, the patient must also follow a low-salt diet and, when possible, recumbency [10].

## Conclusions

This case highlights the etiologies and diagnostic practices associated with idiopathic edema. Thorough patient histories and evaluations are critical for proper diagnosis. Furthermore, safe modifications to edema-related medications and lifestyle changes have been shown to contribute to positive outcomes.
